# Supplementary material for: Chagas disease is related to structural changes of the gut microbiota in adults with chronic infection (TRIPOBIOME Study)
Source: PLoS Negl Trop Dis. 2023 Jul 21;17(7):e0011490. doi: 10.1371/journal.pntd.0011490 (PMC10395948; doi:10.1371/journal.pntd.0011490)
Supplement: S1 Table — (DOCX) [file pntd.0011490.s001.docx]

**Table S1. Baseline characteristics of study population according to treatment**

|  | **No treatment**  **(n=31)** | **Treatment with**  **benznidazole (n=29)** | **Total**  **(n=60)** | **p value** |
| --- | --- | --- | --- | --- |
| Median age (IQR) | 47 (38-57) | 50 (45-56) | 49 (42-56) | 0.44 |
| Sex (women %) | 24 (77.4) | 24 (82.8) | 48 (80.0) | 0.60 |
| Country of origin |  |  |  | 0.17 |
| Bolivia | 28 (90.3) | 27 (93.1) | 55 (91.7) |  |
| Paraguay | 3 (9.7) | 0 (0) | 3 (5.0) |  |
| Brazil | 0 (0) | 1 (3.4) | 1 (1.7) |  |
| Honduras | 0 (0) | 1 (3.4) | 1 (1.7) |  |
| Educational level |  |  |  | 0.72 |
| No studies | 3 (9.7) | 1 (3.4) | 4 (6.7) |  |
| Primary | 12 (38.7) | 10 (34.5) | 22 (36.7) |  |
| Secondary | 14 (45.2) | 15 (51.7) | 29 (48.3) |  |
| University/professional | 2 (6.4) | 3 (10.3) | 5 (8.3) |  |
| Positive PCR for *T. cruzi** | 11 (39.3) | 4 (18.2) | 15 (30) | 0.11 |

Data are numbers (%) or medians (interquartile range). **Trypanosoma cruzi* PCR result was determined at the baseline visit or within the previous month for 50 patients out of 60 with Chagas disease.
