# Supplementary material for: Chagas disease is related to structural changes of the gut microbiota in adults with chronic infection (TRIPOBIOME Study)
Source: PLoS Negl Trop Dis. 2023 Jul 21;17(7):e0011490. doi: 10.1371/journal.pntd.0011490 (PMC10395948; doi:10.1371/journal.pntd.0011490)
Supplement: S2 Table — (DOCX) [file pntd.0011490.s002.docx]

**Table S2. Dietary data of participants with different visceral involvement of Chagas disease compared to controls.**

|  | **Control**  **(n = 12)** | **Cardiac CD**  **(n = 14)** | **Digestive CD**  **(n = 9)** | **Indeterminate CD**  **(n =20)** | **p value** |
| --- | --- | --- | --- | --- | --- |
| Cereals (g/d) ^(A)^  Pulses (g/d)  Greens and vegetables (g/d) ^(A)^  Fruits (g/d)  Milk products (g/d) ^(A)^  Meat and meat products (g/d) ^(A)^  Fish and fish products (g/d)  Eggs (g/d)  Sugars, sweets, pastries (g/d)  Fat and oils (g/d) ^(A)^  Drinks (g/d)  Prepared and precooked foods (g/d)  Aperitives (g/d)  Sauces (g/d) | 153.8 ± 53.0  14.3 ± 38.1  216.3 ± 137.4  126.7 ± 93.7  297.1 ± 163.1  187.2 ± 49.5  14.3 ± 20.9  14.1 ± 18.8  27.4 ± 17.6  31.3 ± 8.4  1202 ± 619  18.1 ± 31.2  10.3 ± 21.3  5.4 ± 12.0 | 147.7 ± 50.8  38.0 ± 66.5  238.4 ± 132.4  205.7 ± 169.8  269.9 ± 162.8  145.8 ± 69.3  11.2 ± 13.4  21.7 ± 24.8  33.3 ± 24.6  33.8 ± 10.9  1105 ± 403  3.4 ± 6.1  2.6 ± 6.1  12.1 ± 15.9 | 124.4 ± 43.6  25.2 ± 23.3  219.1 ± 109.9  256.6 ± 162.0  296.7 ± 107.2  141.0 ± 78.1  19.4 ± 20.6  17.8 ± 23.8  30.3 ± 28.5  32.0 ± 14.6  1342 ± 437  21.5 ± 26.4  2.4 ± 4.5  9.6 ± 12.2 | 155.2 ± 54.1  13.0 ± 32.4  219.5 ± 107.6  216.2 ± 156.1  247.6 ± 143.9  173.4 ± 81.1  15.4 ± 22.7  25.6 ± 25.6  40.6 ± 36.7  35.0 ± 12.3  1337 ± 642  9.7 ± 19.7  2.3 ± 5.6  27.3 ± 36.7 | 0.493  0.074  0.963  0.312  0.769  0.341  0.732  0.576  0.754  0.820  0.608  0.218  0.972  0.060 |
| Energy (kcal/d)  Energy (%EE) ^(A)^  Proteins (g/d)  Proteins (%TEI) ^(A)^  Carbohydrates (g/d) ^(A)^  Carbohydrates (%TEI) ^(A)^  Fiber (g/d)  Lipids (g/d) ^(A)^  Lipids (%TEI) ^(A)^  SFA (%TEI) ^(A)^  PUFA (%TEI)  MUFA (%TEI) ^(A)^  Omega 3 fatty acids (%TEI)  Alpha linolenic acid (%TEI)  EPA + DHA (mg/d)  Omega 6 fatty acids (%TEI)  Omega 6 (g/d)/Omega 3 (g/d) ratio  Trans fatty acids (%ETI) ^(A)^  Cholesterol (mg/d) ^(A)^  Alcohol (g/d) | 2044 ± 332  95.1 ± 5.8  76.7 ± 15.8  15.1 ± 2.6  225.6 ± 50.1  44.0 ± 5.8  15.0 ± 5.1  88.3 ± 15.5  39.0 ± 4.4  12.5 ± 2.6  7.1 ± 2.4  15.8 ± 4.3  0.56 ± 0.24  0.42 ± 0.11  143.9 ± 111.9  6.5 ± 2.4  13.0±6.5  0.47 ± 0.16  284.7 ± 67.9  1.5 ± 3.9 | 1898 ± 287  95.5 ± 7.5  71.1 ± 15.9  15.0 ± 2.7  208.4 ± 43.8  43.9 ± 6.8  18.5 ± 6.2  82.0 ± 18.0  38.8 ± 5.3  12.1 ± 3.0  6.1 ± 2.5  17.3 ± 2.7  0.59 ± 0.26  0.50 ± 0.26  133.1 ± 74.3  5.4 ± 2.2  9.5±3.3  0.45 ± 0.21  270.4 ± 110.9  0.82 ± 1.9 | 1769 ± 198  95.5 ± 10.0  70.6 ± 10.9  16.0 ± 2.2  176.7 ± 29.7  40.1 ± 6.6  21.3 ± 6.3  81.8 ± 17.7  41.4 ± 5.8  12.4 ± 2.6  5.2 ± 1.7  19.8 ± 4.5  0.67 ± 0.20  0.53 ± 0.16  200.8 ± 167.9  4.4 ± 1.6  6.8±2.4  0.35 ±0.18  261.6 ± 76.3  0.21 ± 0.50 | 2035 ± 445  91.9 ± 10.9  79.8 ± 25.0  15.8 ± 4.1  207.0 ± 52.0  40.9 ± 7.2  18.1 ± 5.4  92.6 ± 27.9  40.7 ± 6.3  11.2 ± 3.3  7.8 ± 2.7  17.9 ± 4.3  0.80 ± 0.49  0.61 ± 0.45  342.3 ± 454.4  6.8 ± 2.7  12.6±11.2  0.37 ± 0.23  301.5 ± 109.8  2.6 ±6.4 | 0.315  0.604  0.627  0.823  0.141  0.345  0.211  0.461  0.605  0.595  0.017  0.160  0.326  0.326  0.782  0.020  0.114  0.416  0.709  0.766 |
| Healthy Eating Index ^(A)^ | 61.1 ± 7.6 | 64.5 ± 12.9 | 66.4 ± 12.1 | 62.9 ± 13.3 | 0.765 |
| Thiamine (%DRI)  Riboflavin (%DRI)  Vitamin B6 (%DRI) ^(A)^  Vitamin B12 (%DRI)  Niacin (%DRI)  Folic acid (%DRI) ^(A)^  Vitamin C (%DRI) ^(A)^  Pantothenic acid (%DRI) ^(A)^  Biotin (%DRI) ^(A)^  Vitamin A (%DRI)  Vitamin D (%DRI)  Vitamin E (%DRI)  Vitamin K (%DRI) | 99.4 ± 30.1  111.7 ± 33.1  155.1 ± 52.0  146.9 ± 64.6  210.6 ± 53.9  46.1 ± 15.0  159.9 ± 78.3  101.7 ± 25.1  72.9 ± 27.7  71.2 ± 40.2  6.6 ± 6.5  124.3 ± 49.2  80.6 ± 67.5 | 104.6 ± 23.6  126.4 ± 42.6  142.9 ± 40.8  155.7 ± 46.4  197.8 ± 40.5  59.0 ± 19.6  196.6 ± 109.1  94.4 ± 19.3  91.8 ± 38.8  109.9 ± 55.3  12.4 ± 10.8  124.4 ± 76.0  128.0 ± 88.0 | 110.5 ± 32.9  123.7 ± 36.0  131.7 ± 35.9  150.9 ± 83.9  198.3 ± 52.2  61.5 ± 14.9  189.2 ± 66.6  91.3 ± 26.1  77.5 ± 54.6  105.1 ± 36.7  7.0 ± 6.2  90.4 ± 26.4  166.4 ± 69.6 | 102.0 ± 28.8  108.3 ± 24.5  135.3 ± 35.7  161.6 ± 76.9  190.2 ± 33.9  54.2 ± 19.2  191.7 ± 94.0  94.8 ±21.7  88.4 ± 39.6  75.3 ± 37.3  12.7 ± 15.1  141.1 ± 63.9  118.2 ± 70.9 | 0.818  0.619  0.517  0.747  0.708  0.191  0.732  0.744  0.592  0.058  0.198  0.117  0.031 |
| Calcium (%DRI) ^(A)^  Phosphorus (%DRI) ^(A)^  Magnesium (%DRI) ^(A)^  Iron (%DRI)  Zinc (%DRI) ^(A)^  Iodine (%DRI) ^(A)^  Selenium (%DRI) ^(A)^ | 59.9 ± 23.2  176.3 ± 50.3  63.8 ± 15.1  102.4 ± 46.7  65.0 ± 13.3  50.4 ± 22.5  140.3 ± 49.4 | 57.2 ± 24.8  172.4 ± 48.4  71.7 ± 21.3  102.6 ± 37.0  69.9 ± 16.8  50.3 ± 18.3  119.1 ± 32.2 | 56.5 ± 14.8  175.1 ± 29.6  74.6 ± 19.5  98.2 ± 25.5  69.4 ± 12.5  45.7 ± 13.2  134.6 ± 24.0 | 52.8 ± 17.7  168.1 ± 40.3  70.2 ± 19.2  97.1 ± 28.6  72.4 ± 17.2  48.9 ± 19.8  122.9 ± 32.5 | 0.811  0.955  0.594  0.979  0.642  0.939  0.400 |

Results are presented as mean ± SD. (A): p-value calculated with one-way ANOVA. Other variables: p-value calculated with Kruskal-Wallis test.

food = g edible per day; EE: Energy expenditure; TEI: Total energy intake; SFA: Saturated fatty acids; PUFA: Polyunsaturated fatty acids; MUFA: Monounsaturated fatty acids; EPA: eicosapentanoic acid; DHA: docosahexaenoic acid; DRI: Dietary Reference Intakes.

Bonferroni Post Hoc Test (digestive-indeterminate: PUFA (%TEI) 0.023; digestive-indeterminate: Omega 6 fatty acids (%TEI) 0.032; digestive-control: Vitamin K (%DRI) 0.019).
