# Supplementary material for: Chagas disease is related to structural changes of the gut microbiota in adults with chronic infection (TRIPOBIOME Study)
Source: PLoS Negl Trop Dis. 2023 Jul 21;17(7):e0011490. doi: 10.1371/journal.pntd.0011490 (PMC10395948; doi:10.1371/journal.pntd.0011490)
Supplement: Attachment — Submitted filename: Response to reviewers.docx [file pntd.0011490.s003.docx]

**RESPONSE TO REVIEWERS**

**Reviewer #1: In this manuscript, Pérez-Molina et al performed fecal microbiome characterization of Chagas disease patients and controls. Overall, this is a well-designed study that uses appropriate methods and controls. A particular strength of this study is the dietary questionnaire, which enables the exclusion of dietary confounders. However, authors over-interpret minor differences, and make statements that are not supported by the data. Specifically, I have the following major concerns:**

**1. PLS-DA is prone to over-fitting. Authors should provide metrics to confirm that this is not the case here (explained variation (R2) and predictive ability (Q2)).**

Thank you for your insightful comments about the potential for overfitting in our PLS-DA model. We appreciate the importance of ensuring that our model generalizes well to unseen data. To mitigate the risk of overfitting, we employed several strategies:

Firstly, we were cautious about the complexity of our model. By tuning the number of latent components, we made certain that our model was not overly complex, thereby further reducing the risk of overfitting. For this, we first performed a preliminary Principal Component to evaluate the explained variance per component. As can be seen in the **Figure 1** below, the first two components explain most of the variance observed in the microbiome dataset.

**Figure 1**. Barplot of explained variance in the microbiome dataset per principal component.

This analysis prompted us to fit a simplified sPLS-DA model, restricted to the first two components, which was the one shown in the previous version of the manuscript. As can appreciate below, the disease group explains in the Y axis a 6% of the variance.

**Figure 2**. Initial sPLS-DA model (shown in the previous version of the manuscript)

Following this Reviewer’s comment, to further the risk of overfitting we have optimized the sPLS-DA model shown in Figure 2 by training the model using cross-validation. This technique is a widely recognized and effective way to prevent overfitting by providing an unbiased estimate of model prediction performance. This strategy allowed us to evaluate the classification rates for each component. The minimum prediction distance “max.dist” is reached at component 3. Following this analysis, we optimized the number of components in the sPLS-DA model to 3.

**Figure 2**. Initial sPLS-DA model (shown in the previous version of the manuscript)

**Figure 3**. Classification rate for each component in the sPLS-DA analysis. For each component, repeated cross-validation (3 × 3−fold CV) is used to evaluate the sPLS-DA classification performance (OER and BER), for each type of prediction distance; max.dist, centroids.dist and mahalanobis.dist.

Then, we determined the optimal number of variables that should be tested for each component by cross-validation. As can be seen in Figure 3, the optimal number of variables for components 1, 2 and 3 is 20, 6, and 20, respectively.

**Figure 4**. Tuning the number of variables to select with cross-validation.

Finally, we have fitted the optimized sPLS-DA model restricted to the first three components and with the number of variables selected as defined above. As can be seen in the plot projecting the samples onto the first two components of the sPLA-DA subspace that has been updated in the abstract, the Chagas disease condition explains a 5% of the variance in the microbiome dataset.

**Figure 5**. Explained variance of microbiota composition in patients with Chagas diseases vs. controls using sPLS-DA modelization.

In response to the query about the predictive performance (Q2) of our model, our bioinformatics tool, mixomics, does not allow for direct calculation of the Q2 statistic for sPLS-DA models. However, an alternative and highly informative measure of model performance is the Area Under the Receiver Operating Characteristic Curve (AUC-ROC). In the context of our binary classification problem (Chagas vs. controls), our model achieved an AUC of 0.95. This indicates a high degree of predictive accuracy, as an AUC of 1 represents a perfect model and an AUC of 0.5 signifies a model that performs no better than random chance. This result suggests that our model possesses a robust predictive capacity.

**Figure 6**. Area under the Receiver Operating Characteristic Curve of the optimized sPLS-DA model for the effect of Chagas disease on the microbiota composition.

We will clarify these points in the revised manuscript to ensure that the steps we took to prevent overfitting, the explained variance of the optimized sPLS-DA model and the predictive performance are clearly understood by all readers

**2. Heat tree analyses are an atypical way to present the data. Authors should provide additional text on figure interpretations, and on the methods used to generate this figure.**

Response: we have included a link to the heat tree code and additional text for interpreting the figure in the methods section.

**3. Authors should also clarify the discrepancies between the differential bacteria displayed on the volcano plot and on the heat tree.**

Response: both the volcano plot and the heat tree are constructed based on the same statistical output, but the algorithm used by R to annotate the dots in the volcano plot randomly omits some points to enhance readability. While the volcano plot offers information on the statistical significance and effect size easier to interpret, the heat tree map offers more phylogenetic information, and the whole list of differential bacteria. In addition, the heat tree is restricted to the statistically significant taxa (adjusted P-value <0.001).

Following this Reviewer’s comment, we have edited the volcano plot to maximize the number of annotated bacteria.

**4. Lines 275-277: this statement is not supported by the data. Indeed, the differences in Enterococcus hirae, Lactobacillus buchneri, and Megamonas between CD and controls appear to be primarily outlier-derived, with group medians and quartile overlapping fully. The differences in Parabacteroides appear more robust.**

Response: Thank you for the insightful comment. Following this concern, we have performed additional non-parametric statistical tests that do not make assumptions about the distribution of the data. The Mann-Whitney U test is based on the ranks of the data rather than their absolute values, which makes it less sensitive to outliers. It essentially compares whether one group tends to have larger values than the other group, so we have applied to this list of bacteria for which we claimed differences in Figure 3. As hinted by the Reviewer, only *Parabacteroides sp*. retained the statistical significance (p=0.038)

Therefore, we have tempered our claims regarding these taxa, acknowledge the possible influence of outliers and emphasized the potential role of *Parabacteroides sp*. as a marker of Chagas disease.

**5. Authors should test whether these outliers come from the six patients with more-severe disease symptoms**

Response: We appreciate the reviewer's comment, and we have analysed specifically such cases. We did not detect that the outliers were related to subjects with the most severe visceral involvement in Chagas disease. The patients most enriched in *E. hirae*, *Lactobacillus spp* and *Megamonas spp* were those with Chagas disease (as opposed to controls), regardless of the form of the disease (indeterminate, cardiac or digestive) and its severity. While those outliers were present in any form of the disease for *E. hirae*, they were more common in the digestive form for *Megamonas spp* and were not present in the digestive form for *Lactobacillus spp*. We have added a comment on this topic in the results section.

**6. Authors use the heatmap in figure 6 to justify a lack of impact of dietary, treatment or sex-based confounders. However, given that the samples also do not cluster by Chagas status, the study may simply be under-powered to address confounders.**

Response: While we have acknowledged that the study is exploratory when interpreting the data and in the limitations section, we had also performed a multivariate analysis (PERMANOVA) to address the potential confounding effect of sex and Chagas treatment on the overall microbiota structure (beta diversity), as we stated in the results section. The PERMANOVA analysis did not show a significant effect for sex or treatment on microbiota composition. Following this Reviewer’s comment, we have also performed pairwise comparisons for alpha diversity by sex (figure 7) and Chagas treatment group (figure 8), and no differences are appreciated.

**Figure 7**. **Differences in alpha diversity metrics (Simpson and Shannon indexes) by sex group.** No differences were found.

**Figure 8**. **Differences in alpha diversity metrics (Simpson and Shannon indexes) by treatment group.** No differences were found.

In the heatmap, the lack of clustering by these factors further diminishes the concern of a potential confounding effect. While we could not perform a similar analysis by dietary component, given the high dimensional nature of the dietary information collected, the lack of substantial differences between groups in the dietary information analyzed (Table 2 and Table 3 in the manuscript) does not suggest a potential confounding effect of diet in our analysis.

**7. Authors under-used their metagenomics data by merely performing species-level analyses. Important analyses should also investigate whether there are specific bacterial genes or pathways that differ between groups.**

Response: The point raised by the reviewer is well-taken. The shotgun metagenomic analysis allows us to investigate bacterial pathways differing between groups. This analysis will be the object of subsequent work to explore the correlations between bacterial functions and proteomic analysis in plasma. We have not yet entirely performed such analysis, which is very time-consuming, and we decided to keep that data for subsequent work.

**8. Authors fail to cite recent work already published on this topic using 16S sequencing (de Souza-Basqueira et al, doi: 10.3389/fcimb.2020.00402). A comparison of findings from the current study vs this prior publication should be performed. This also leads to the incorrect statement of novelty by the authors at lines 387-389.**

Response: We appreciate the reviewer's feedback regarding the citation of recent work (de Souza-Basqueira et al., doi: 10.3389/fcimb.2020.00402) and the need for a comparison between the findings of the current study and that prior publication. We have revised the manuscript to include a discussion and performed a comparison between their findings and the results presented in our study. We have also addressed the criticism raised at lines 387-389, where we initially made a statement of novelty.

**9. Lines 304-305: “we found a small impact of this parasitosis on bacterial richness”. Richness differences did not reach statistical significance and thus should not be stated as a finding.**

Response: Response: We agree with the Reviewer. We have modified this sentence to state that we found no differences in alpha diversity.

**10. Authors should specify whether beta-diversity differences were observed between the different CD forms**

Response: To give consideration to this Reviewer’s comment, we have performed a PERMANOVA analysis to test differences in beta diversity across groups. The analysis did not show a significant effect of the different Chagas disease categories. Accordingly, we have added the following statement to the results:

*“A PERMANOVA analysis to test differences in beta diversity between Chagas disease groups did not suggest a significant effect (R2 = 0.047, P = 0.153).”*

**Minor concerns:**

**1. Line 223-225: this statement is not supported by Table S2, since significant differences in PUFA and Omega 6 fatty acids were observed.**

Response: We agree with the reviewer on this difference that we have highlighted and commented on in the text. In addition, we have added information on vitamin and mineral intakes in case of further differences (Table 3), with small imbalances in vitamin K intakes having been detected. All these data have been added to the results and commented on in the discussion.

**2. Line 223 (“data not shown”). Data should be provided in SI.**

Response: We have added this information (Table 3).

**3. There are two color legends in Figure 4b.**

Response: Thank you for noting this. We have amended the figure.

**4. Line 113-118: the details on REDcap are unnecessary and irrelevant to the study’s findings**

Response: We have simplified the description about REDcap in the methods section

**5. Given that E. hirae is rarely associated with humans, and yet is found at relatively high abundance in both CD and controls in this study, authors should verify their taxonomic assignments, before making this large interpretation.**

Response: We have checked the results of *E. hirae* using centrifuge and we obtained similar results. Please see attached Figure.

**6. Lines 380-385: italicize Prevotella.**

Response: We have italicised it.
